# Retraction: The N-Localizer and Volume Imaging

**DOI:** 10.7759/cureus.r6

**Published:** 2016-06-23

**Authors:** Russell A Brown

**Affiliations:** 1 Principal Engineer, Retired, Palo Alto, USA

This article (Brown R A (December 03, 2014) The N-Localizer and Volume Imaging. Cureus 6(12): e232. doi:10.7759/cureus.232) is being retracted because it has been replaced by an updated and improved article with additional information http://www.cureus.com/articles/3193 Brown R A (October 12, 2015) The Mathematics of Four or More N-Localizers for Stereotactic Neurosurgery. Cureus 7(10): e349. doi:10.7759/cureus.349 http://www.cureus.com/articles/3144.

